# Determining the Online Measurable Input Variables in Human Joint Moment Intelligent Prediction Based on the Hill Muscle Model

**DOI:** 10.3390/s20041185

**Published:** 2020-02-21

**Authors:** Baoping Xiong, Nianyin Zeng, Yurong Li, Min Du, Meilan Huang, Wuxiang Shi, Guojun Mao, Yuan Yang

**Affiliations:** 1 College of Physics and Information Engineering, Fuzhou University, Fuzhou City 350116, Fujian Province, China; xiongbp@fjut.edu.cn (B.X.); hml19940515@icloud.com (M.H.); shiwuxiang3@163.com (W.S.); 2 Department of Mathematics and Physics, Fujian University of Technology, Fuzhou City 350118, Fujian Province, China; maximmao@hotmail.com; 3 Department of Instrumental and Electrical Engineering, Xiamen University, Fujian 361005, China; 4 Fujian Key Laboratory of Medical Instrumentation & Pharmaceutical Technology, Fuzhou University, Fuzhou City 350116, Fujian Province, China; liyurong@fzu.edu.cn; 5 Fujian provincial key laboratory of eco-industrial green technology, Wuyi University, Wuyishan City 354300, Fujian Province, China; 6 Department of Physical Therapy and Human Movement Sciences, Northwestern University, Chicago, IL 60208, USA

**Keywords:** artificial neural network, joint moment prediction, extreme learning machine, Hill muscle model, online input variables

## Abstract

*Introduction*: Human joint moment is a critical parameter to rehabilitation assessment and human-robot interaction, which can be predicted using an artificial neural network (ANN) model. However, challenge remains as lack of an effective approach to determining the input variables for the ANN model in joint moment prediction, which determines the number of input sensors and the complexity of prediction. *Methods*: To address this research gap, this study develops a mathematical model based on the Hill muscle model to determining the online input variables of the ANN for the prediction of joint moments. In this method, the muscle activation, muscle-tendon moment velocity and length in the Hill muscle model and muscle-tendon moment arm are translated to the online measurable variables, i.e., muscle electromyography (EMG), joint angles and angular velocities of the muscle span. To test the predictive ability of these input variables, an ANN model is designed and trained to predict joint moments. The ANN model with the online measurable input variables is tested on the experimental data collected from ten healthy subjects running with the speeds of 2, 3, 4 and 5 m/s on a treadmill. The variance accounted for (VAF) between the predicted and inverse dynamics moment is used to evaluate the prediction accuracy. *Results*: The results suggested that the method can predict joint moments with a higher accuracy (mean VAF = 89.67±5.56 %) than those obtained by using other joint angles and angular velocities as inputs (mean VAF = 86.27±6.6%) evaluated by jack-knife cross-validation. *Conclusions*: The proposed method provides us with a powerful tool to predict joint moment based on online measurable variables, which establishes the theoretical basis for optimizing the input sensors and detection complexity of the prediction system. It may facilitate the research on exoskeleton robot control and real-time gait analysis in motor rehabilitation.

## 1. Introduction

Human joint moment prediction is crucial to rehabilitation evaluation [1,2,3], athlete training evaluation [4,5,6], prosthesis and orthosis design [7,8,9], intramedullary device design [10,11,12] and human-robot interaction [13,14,15,16,17,18,19,20,21]. The precise prediction of joint moment can be fulfilled by the use of instrumented implants [22] which measures the relevant parameters of joint load in real time. However, this approach is not always feasible since only few people (likely those suffering from musculoskeletal deficits) have implants.

Although computational models can serve as alternative methods for joint moment prediction when the implants are not available, they face a challenge of eliminating the measurement error. This is due to the individual differences in the anatomical and functional characteristics of the musculoskeletal system [22]. Furthermore, the joint moment is not easily measured in real time. Previous studies [23,24,25,26] indicated that this challenge may be addressed by using the artificial neural network (ANN) model, because of its excellent adaptive ability to individual characteristics [27,28]. For example, Uchiyama et al. [29], used an ANN model to predict the elbow joint moment with the inputs of EMG signals, elbow and shoulder joint angles, while Luh et al. [30], and Song and Tong [31] utilized an ANN model with EMG signals, elbow joint angle and angular velocity for the same purpose. Hahn [32] intelligently predicted the isokinetic knee extensor and flexor moment with the inputs of EMG signals, gender, age, height and body mass. Ardestani et al. [33], combined the EMG signals and ground reaction force (GRFs) with ANN model to study the lower limbs’ joint moment. Recently, Xiong et al. [34], used the optimized EMG signals and joint angles as the inputs of ANN model to calculate the lower extremity joint moment.

As listed above, different studies used different input variables in their ANN models to predict joint moments. However, the number of input variables determines the number of sensors and the complexity of the system. It is yet to develop a mathematical model to determine the optimal online measurable input variables. This model will provide a theoretical basis for designing a system with few sensors and high accurate of joint moment prediction. Therefore, the purpose of this study is to introduce a novel method for determining the online measurable input variables for human joint moment intelligent prediction.

In this method, musculoskeletal geometry [35,36] comprised of Hill muscle models [37,38] are utilized for representing the muscle mechanical response. Furthermore, the input variables to predict joint moment based on the Hill muscle model includes four time-varying variables: the muscle activation, muscle-tendon moment arm, velocity and length are found [39], that generally cannot be measured online in vivo. Thus, a surrogate model is built for each tested muscle to convert these four input variables to the online measurable variables, i.e., muscles EMG, the muscle actuates joints’ angles and angular velocities. 

To test the predictive ability of the online measurable input variables, a commonly used ANN model, i.e., Extreme Learning Machine (ELM), is designed and trained to predict joint moments. The ELM is a feedforward ANN [40], which has a much lower computational cost than traditional machine learning algorithms, especially for the single hidden layer mode [41,42,43]. The method is tested on the experimental data of ten healthy male subjects running at different speeds, i.e., 2, 3, 4 and 5 m/s on a treadmill. The ELM predictions are validated against inverse dynamics and compared with those obtained by jack-knife cross-validation with other online measurable variables as inputs [29,30,31,34].

## 2. Materials and Methods

### 2.1. Experimental Data

The lower limbs’ kinematics and dynamics experimental data of ten healthy male subjects (height 1.77 ± 0.04 m, age 29 ± 5 years, mass 70.9 ± 7.0 kg) was obtained from an open database (https://simtk.org/projects/nmbl_running; accessed on, 18 October 2019). In the experiment, the motion data, EMG signals and ground reaction force were measured, while the subjects ran at different speeds of 2, 3, 4 and 5 m / s on the treadmill. At least six gait cycles were recorded for each speed. The EMG signals included gluteus medius, rectus femoris, gluteus maximus, vastus lateralis, biceps femoris long head, vastus medialis, tibialis anterior, soleus, gastrocnemius medialis and gastrocnemius lateralis. All the EMG signals were rectified, filtered and normalized. The motion and force data were filtered accordingly. A complete description of these data can be found in [44].

After obtaining the experimental data, all the ten subjects’ moment of ankle plantar-dorsiflexion, knee flexion-extension, hip adduction-abduction and hip flexion-extension are firstly calculated by using the inverse dynamics method [45] with opensim software, then the moment, force, motion and EMG signals are resampled to obtain 101 time points of each gait cycle. All the inverse dynamics moment will be used as the target value of the ANN model’s training samples.

### 2.2. Determination of Online Measurable Variables

**Figure 1:**
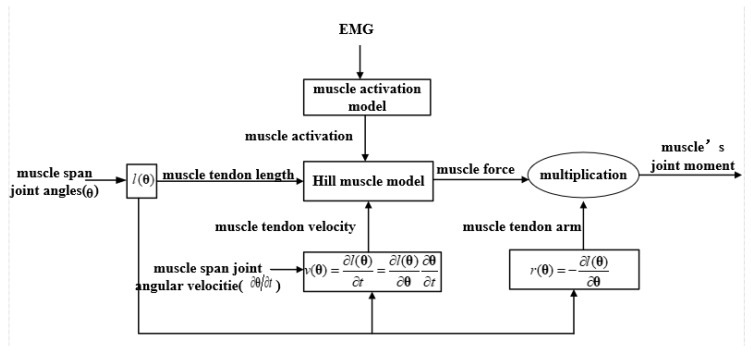
Data processing pipeline of the method based on Hill muscle model, where l(θ) is a polynomial function of the muscle spans joint angles.

In order to obtain the online measurable input variables, the Hill muscle model [37,38] and musculoskeletal geometry [35] is used to establish a mathematical model of input-output relation for joint moment prediction. The data processing pipeline is shown as Figure 1.

**Figure 2:**
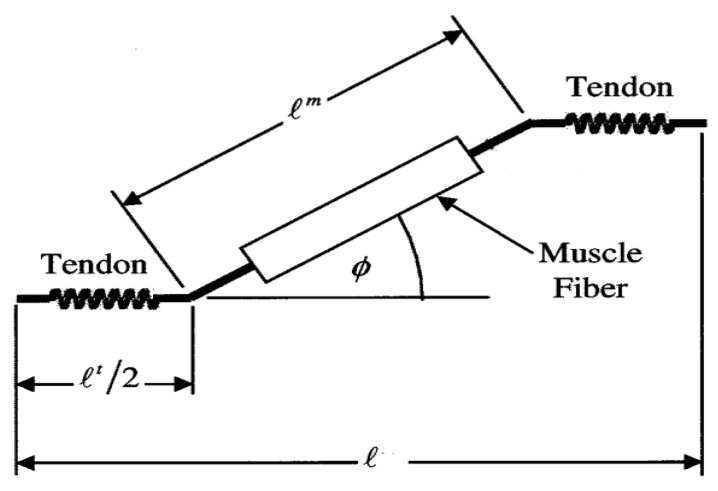
A diagram of muscle-tendon unit that shows the relationship of muscle-tendon length, muscle fiber length, tendon length, pennation angle. Where l is the muscle-tendon length, $l^{m}$ is the muscle fiber length, $l^{t}$ is the tendon length, ϕ is the pennation angle.

In the Hill muscle model, the muscle moment about the spanned joint [46] is indicated by:

(1) $$M=r\cdot F_{o}^{M}\cdot [a(emg(t-d))\cdot f_{l}(\frac{l-l_{s}^{T}}{l_{o}^{M}\cos\phi })\cdot f_{v}(\frac{v}{10\cdot l_{o}^{M}})+f_{p}(\frac{l-l_{s}^{T}}{l_{o}^{M}\cos\phi }))]\cos(\phi )$$

where M and r are the muscle moment and moment arm about the joint it actuates, $F_{O}^{M}$ is muscle’s peak isometric force, a() is the muscle’s activation which can be calculated as a function of EMG data, t is the time, d is the electromechanical delay, v and l are muscle-tendon velocity and length, ϕ is pennation angle of the muscle, $l_{o}^{M}$ is the optimal fiber length and $l_{S}^{T}$ is the tendon slack length. The relationship of muscle-tendon length, muscle fiber length, tendon length, pennation angle can be seen in Figure 2. $f_{v}()$, $f_{l}()$ and $f_{P}()$ represent muscle force-velocity, active force-length and passive force-length curve. $F_{o}^{M}$, d, ϕ, $l_{s}^{T}$ and $l_{o}^{M}$ are assumed to remain constant for the individual. l, v and r are time variables that can be calculated as polynomial functions of joint angles and angular velocities with the same constant coefficients [47,48]. When θ is the muscle spans joint angles, those time variables can be expressed as follows:

(2) l(t)=l(θ)

(3) $$v(t)=\frac{\partial l(t)}{\partial t}=\frac{\partial l(\theta )}{\partial t}=\frac{\partial l(\theta )}{\partial \theta }\;\frac{\partial \theta }{\partial t}=v(\theta ,\overset{•}{\theta })$$

(4) $$r(t)=-\frac{\partial l(\theta )}{\partial \theta }=r(\theta )$$

where θ(t) and $\overset{•}{\theta }(t)$ are the muscle spans joint angles and angular velocities; l(θ) is muscle-tendon length which is polynomial functions of the muscle spans joint angles;$v(\theta ,\overset{•}{\theta })$ is muscle-tendon velocity which is the first derivative of l(θ) with respect to time t; r(θ) is muscle-tendon moment arm which is the first derivative of l(θ) with respect to θ. The sign of the variable is used to determine the direction of the moment.

From Equations (1)–(4), the muscle moment about the spanned joint can be calculated as a function of the muscle’s EMG signal, and the muscle actuates joints’ angle and angular velocity (Figure 1):

(5) $$M(emg,\theta ,\overset{•}{\theta })=r(\theta )\cdot F_{o}^{M}\cdot [a(emg(t-d))\cdot f_{l}(l(\theta ))\cdot f_{v}(v(\theta ,\overset{•}{\theta }))+f_{p}(l(\theta ))]\cos(a)$$

where d is an electromechanical delay, and its value is generally 10-100ms [49]. From Equations (1)–(5) the j-th joint moment is represented by the following equation:

(6) $$M^{j}=\overset{m}{\underset{i=1}{\sum }}M(emg(i),\theta (i),\overset{•}{\theta }(i))$$

where m is the number of muscles associated with the joint moment.

It can be seen from Equation (6) that the online measurable input variables for the human joint moment prediction are joint moment-associated muscles’ EMG signals, and their muscles actuates joints’ angles and angular velocities.

### 2.3. The Designed ANN

**Figure 3:**
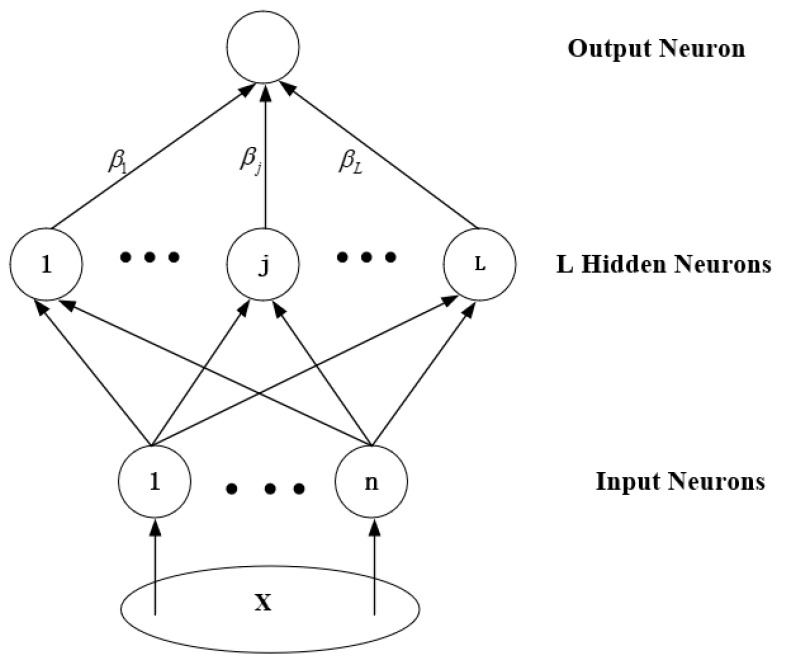
Structure of the designed ELM.

To confirm the predictive effect of the online measurable input variables, the ELM is designed and trained as the ANN model to predict joint moments, which is a feedforward ANN algorithm [40]. It can be seen from Equation (6) that different joint moments correspond to different inputs which is not suitable to use the multi-output ANN model, so the ELM only has one output neuron. Its structure is generally shown as Figure 3, which is divided into an input layer, a hidden layer and an output layer. Its expression is provided as follows:

(7) O=βg(W·X+b)

where X is the input, O is output, $W=[W_{1},W_{2},\cdots ,W_{L}]$ is the matrix of input-to-hidden-layer weights, $\beta =[\beta_{1},\beta_{2},\cdots ,\beta_{L}]$ is the matrix of hidden-to-output-layer weights, $b=[b_{1},b_{2},\cdots b_{L}]$ is the matrix threshold of the hidden node and g() is the activation function. The distinguishing feature of ELM from the traditional feedforward neural network is that W and b are randomly selected and does not need to be adjusted during the training process, and β are calculated in the training process [45]. The feature makes the process of determining network parameters without iterations, reduces the adjustment time of network parameters, and greatly improves the learning speed. The ELM is widely used in regression analysis and classification [41,50].

The ELM is trained to predict four DOFs’ moment in the right leg: ankle plantar-dorsiflexion (Ankle PDF), knee flexion-extension (Knee FE), hip adduction-abduction (Hip AA) and hip flexion-extension (Hip FE), and the inverse dynamics moment is used as the target value of the training sample. It can be seen from Table 1 with Equation (6) that the input variables of Hip FE’s joint moment prediction contains the EMG signals of four muscles and three joint angles and angular velocities. There are 10 input variables in total.

### 2.4. Prediction Evaluation

**Figure 4:**
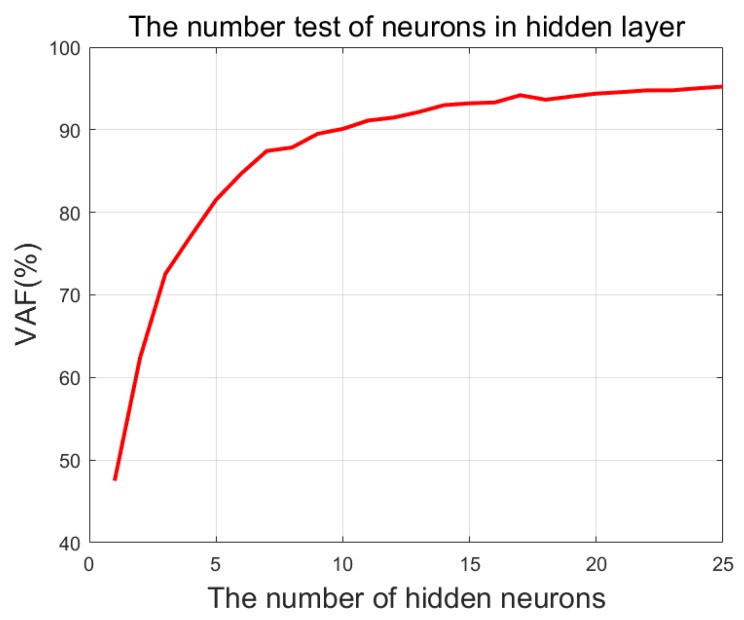
The ten subject’s average predict accuracy evaluated by the variance accounted for (%) with the increase of neurons.

Considering that Equation (6) is obtained under the assumption that $F_{o}^{M}$ (muscle’s peak isometric force), d (the electromechanical delay), ϕ (pennation angle of the muscle), $l_{s}^{T}$ (the tendon slack length) and $l_{o}^{M}$ (the optimal fiber length) are remain constant for the individual, which is not suitable for training multiple subjects at a time, so per ELM only trains one joint moment of a subject. A generic three-layer ELM is designed and trained using two strategies for evaluating the generalization ability of the method at two different levels: (1) training with all four speeds (level 1) and (2) training only with the three low speeds (2, 3 and 4 m/s) (level 2). During the supervised training, the inverse dynamics moment is used as the target value of the training samples. The variance accounted for (VAF) [51] is used to evaluate the accuracy of the ELM, its expression is as follows:

(8) $$\mathrm{VAF}=[1-\frac{\mathrm{var}(\hat{y}-y)}{\mathrm{var}(y)}]\times 100\%$$

where y is the inverse dynamics moment and $\hat{y}$ is predicted joint moment. For each speed, six gait cycles (6 × 101 = 606) are selected for training and testing. Since a complete gait cycle data may contain all gait features at the current speed, training and testing must take the whole gait cycle as input or it is easy to cause feature loss to make the prediction result unstable. Therefore, the data set is smaller, a greater percentage of 30% as testing data set and 70% as training data set must be used to train and test the ELM, so four (6 × 0.7 = 4.2) gait cycles (4 × 101 = 404 time points) data are randomly selected from each tested speed for training, and the remaining two (6 × 3 = 1.8) gait cycles (202 time points) for testing. Then, in order to set the appropriate number of neurons in the hidden layer for better prediction effect, an experiment is done to observe the relationship between the number of neurons in the hidden layer and the prediction accuracy. In the experiment, four gait cycles data are selected from each speed for training, and two gait cycles for testing. The ten subjects’ average predicted accuracy evaluated by the VAF (%) are shown as Figure 4. 

It can be seen from Figure 4 that the value of VAF increased rapidly with the increase of neurons at the beginning, but the value of VAF slowed down when the number of neurons exceeded 10. Considering the structural complexity of ELM and the time cost for training, the number of neurons in the hidden layer is set to 20.

## 3. Results

**Figure 5:**
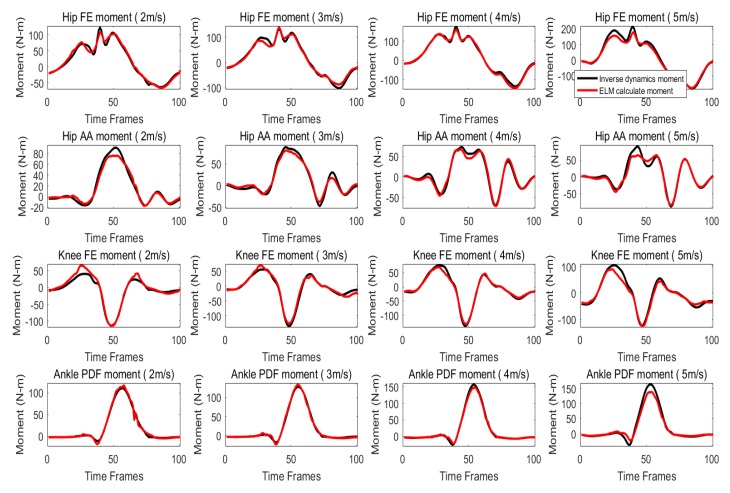
Joint moment prediction of a typical subject at each speed when all four speeds are used for training (level 1).

When training with all four speeds (level 1), the trained ANN model is used to predict the lower limbs’ joint moment of all subjects at different speeds. Joint moment prediction of a typical subject at each speed are shown in Figure 5. As shown, the general pattern of lower limb joint moment can be predicted well at each speed. Comparing with inverse dynamics moment, there only have some difference in minimum and maximum values of waveforms (cross-correlation coefficient > 0.987). The VAF of the predicted joint moment for Ankle PDF, Knee FE, Hip FE and Hip AA at level 1, with the mean VAF (± standard deviation) of 97.15±0.99%, 94.23±2.99%, 95.39±3.62% and 95.01±7.46% as shown in Table 2.

**Figure 6:**
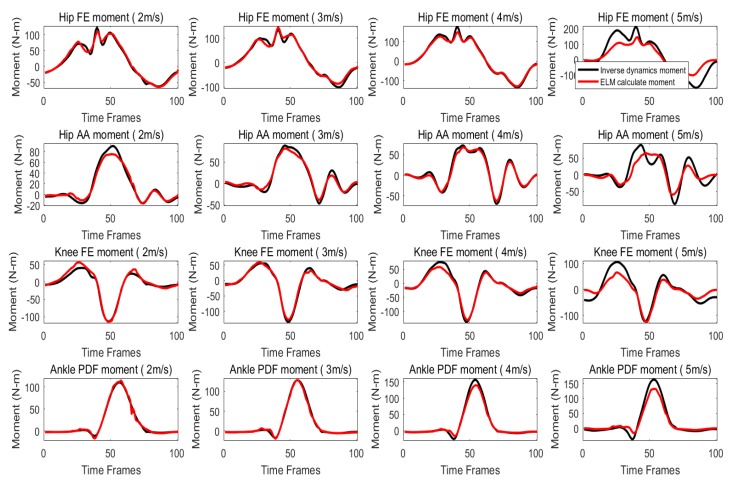
Joint moment prediction of a typical subject at each speed when only the three low speeds are used for training (level 2).

When training with the three low speeds (level 2), the trained ANN model is also used to predict the lower limbs’ joint moment of all objects at different speeds. Joint moment prediction of a typical subject at each speed are shown in Figure 6. As shown, the errors between the predicted and inverse dynamics moment were slightly increased, when compared to the corresponding errors at level 1 (cross-correlation coefficient > 0.984), especially the speed of 5m/s. The VAF of the predicted joint moment for Ankle PDF, Knee FE, Hip FE and Hip AA at level 1, with the mean VAF (± standard deviation) of 94.31±7.13, 93.04±3.62, 92.08±2.93% and 89.95±2.31% as shown in Table 3. 

In order to examine generalizability over multiple conditions, a more exhaustive validation of the test result data is conducted using jack-knife cross-validation [52] which all cross-validation subsets consist of only one data set each. In the jack-knife cross-validation, six gait cycles at each speed are taken as one data set, and there are four data sets in total. In each test, three data sets are selected as training sets and one data set as test set, and their average VAF of ten subjects ‘predicted joint moment for Ankle PDF, Knee FE, Hip FE and Hip AA are shown in Table 4. As shown in Table 4, the obtained results have little difference from level 2.

**Figure 7:**
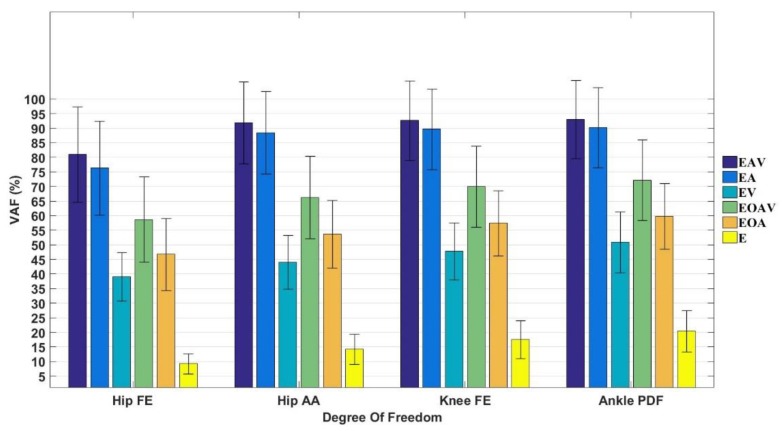
Comparison of performance by jack-knife cross-validation for several combination of inputs: EAV = relevant muscles’ EMG, and their muscles actuate joints’ Angles and angular Velocities; EA = relevant muscles’ EMG and their muscles actuate joints’ Angles; EV = relevant muscles’ EMG and their muscles actuate joints’ Angles; EJAV= relevant muscles’ EMG, the Joint’s Angle and Angular velocity; EJA = relevant muscles’ EMG and the Joint’s Angular velocity; E = relevant muscles’ EMG signals.

Furthermore, the method (EAV) is compared with other combination of inputs using jack-knife cross-validation by VAF (Figure 7). 

They are five different inputs as following: (1) Relevant EMG signals and their muscles actuate joints’ Angles (EA); (2) Relevant EMG signals and their muscles actuate joints’ angular Velocities (EV); (3) Relevant muscles’ EMG signals, the Joint’s Angle and angular Velocity (EJAV); (4) Relevant muscles’ EMG signals and the Joint’s Angle (EJA); (5) Relevant muscles’ EMG signals as inputs (E). The relevant muscles’ EMG signals means that the joint moment-associated muscles’ EMG signals. Take EAV ($\bar{\mathrm{VAF}}=89.67\pm 5.56$%) as reference and compare with the above inputs respectively, It can be seen that the $\bar{\mathrm{VAF}}$ of the moment predicted by the EA ($\bar{\mathrm{VAF}}=86.21\pm 6.60$%), EV($\bar{\mathrm{VAF}}=45.48\pm 5.08$%), EJAV($\bar{\mathrm{VAF}}=66.80\pm 5.91$%), EJA($\bar{\mathrm{VAF}}=54.41\pm 5.70$%), and E($\bar{\mathrm{VAF}}=15.39\pm 4.81$%) are almost reduced by 3.85%, 49.27%, 25.50%, 39.31% and 82.83%.

## 4. Discussion and Conclusions

This study demonstrated that the ELM with the online measurable input variables could be used as a real-time surrogate model to predict joint moments under different gait speeds. Compared with the previous studies [29,30,31,32,33,53,54,55], this research extends our knowledge by establishing the mathematical model of input-output relation in the human joint moment prediction based on the Hill muscle model. The online measurable input variables are obtained for the ANN model. It does not need ground reaction force and marker trajectories which increases the number of input sensors and the complexity of prediction. The novel method has high prediction accuracy with $\bar{\mathrm{VAF}}=96.07\pm 3.484$%. Thus, the proposed method is suitable for online rehabilitation assessment and human-robot interaction which need to obtain joint moment in real time.

It can be seen from Equations (1)–(6) that the muscles actuate joints are very limited, while inertial magnetic measurement systems are good at measuring the limited joints’ angles and angular velocities [56], so unlike previous computational models, such as inverse dynamics [57,58] and EMG-driven models [39,46,59], the method can online predict joint moment without essential 3D motion capture and complicated calculation, which make the hospitals and laboratories to predict joint moments without site requirements, even in a free state. It can also adapt to the individual differences in the process of training, and does not need the musculoskeletal model or the scaling of specific objects, thereby reducing the error caused by individual differences. Furthermore, the training time is less than one second.

Compared level 2 with level 1 and the jack-knife cross-validation results (Table 4), the results suggest that the proposed method has a good generalization ability. Thus, in practice, a reduced amount of training data can be used when a large amount of data is not available. It can be seen from Figure 7 that EAV has the best prediction results in all joints compared with other inputs, which verifies the accuracy of the method proposed in this paper. Comparing our method with EA, the latter’s $\bar{\mathrm{VAF}}$ only reduced by 3.85%. Thus, it can be concluded that the effect of angular velocities on joint moment prediction is relatively small. Comparing the method with E, the latter’s $\bar{\mathrm{VAF}}$ reduced by 82.83%. This indicates that: (1) the EMG value alone cannot represent the value of the joint moment [60], and (2) the joint angle has a great influence on the joint moment prediction. From Figure 7, It can also be found that the EJAV has good prediction results, so it can be concluded that the effect of the joint moment’s angle and angular velocity on joint moment prediction is very important. This is the reason why the musculoskeletal model use joint’s angles and angular velocities as inputs to calculate joint moments. As the ANN model can adapt to the individual differences in the process of training and the muscle model is applicable to all muscles of any human body whether male or female, old or young and health or not, so the proposed method can also be applied to other joints of any human body theoretically.

It should be mentioned that the current study has some limitations. Firstly, there are only 10 muscles’ EMG data of the right leg used in the method, which can’t represent all muscles associated with the joint. our approach will be developed in a larger set in the future. Secondly, the gait patterns in the experimental only include run gait patterns, which is very limited. In the future study, more gait data will be collected, such as squatting, cutting and so on. Finally, the sample is only composed of young male subjects with similar anthropometry and age, which cannot ensure the diversity of the training samples. Data samples from different groups of people will be collected in the future, such as children, old people, women, patients and so on.

## Figures and Tables

**Table 1:** The list of EMG signal sources and their muscle actuates.

EMG Signal Source	Actuates
Gluteus maximus	Hip AA, Hip FE,
Gluteus medius	Hip AA, Hip FE
Biceps femoris long head	Knee FE, Hip AA, Hip FE
Rectus femoris	Knee FE, Hip AA, Hip FE
Vastus medialis	Knee FE
Vastus lateralis	Knee FE
Gastrocnemius lateral	Knee FE, Ankle PDF, Ankle IE
Gastrocnemius medial	Knee FE, Ankle PDF, Ankle IE
Tibialis anterior	Ankle PDF, Ankle IE
Soleus	Ankle PDF, Ankle IE

**Table 2:** Joint moment prediction performances for level 1, evaluated by VAF (%).

Participants	Hip FE	Hip AA	Knee FE	Ankle PDF
subject 1	97	94.50	96.47	98.11
subject 2	96.98	95.80	96.90	97.61
subject 3	94.85	87.02	86.69	73.89
subject 4	97.69	96.17	98.20	98.27
subject 5	96.86	92.15	95.12	96.94
subject 6	96.37	93.58	94.65	96.40
subject 7	97.78	96.74	95.46	96.65
subject 8	97.88	96.54	97.62	98.42
subject 9	98.15	96.46	98.02	96.22
subject 10	97.94	93.37	95.73	97.62
mean	97.15	94.23	95.39	95.01
Std	0.99	2.99	3.62	7.46

**Table 3:** Joint moment prediction performances for level 2, evaluated by VAF (%).

Participants	Hip FE	Hip AA	Knee FE	Ankle PDF
subject 1	88.31	94.06	93.04	97.50
subject 2	88.09	94.26	93.48	96.82
subject 3	89.80	86.52	84.55	74.20
subject 4	92.07	94.84	97.58	98.06
subject 5	85.36	89.84	92.40	95.92
subject 6	89.17	90.44	92.73	95.66
subject 7	92.14	94.56	92.68	95.85
subject 8	92.81	94	96.20	97.72
subject 9	91.67	93.43	96.49	94.89
subject 10	90.08	88.81	91.32	96.51
mean	89.95	92.08	93.04	94.31
Std	2.31	2.93	3.62	7.13

**Table 4:** Joint moment prediction performances for jack-knife cross-validation, evaluated by VAF (%).

Participants	Hip FE	Hip AA	Knee FE	Ankle PDF
mean	81.07	91.88	92.68	93.09
Std	16.37	14.10	13.67	13.42

## Founding

This research received no external funding.
